# Supplementary material for: Paf1 and Ctr9 subcomplex formation is essential for Paf1 complex assembly and functional regulation
Source: Nat Commun. 2018 Sep 18;9:3795. doi: 10.1038/s41467-018-06237-7 (PMC6143631; doi:10.1038/s41467-018-06237-7)
Supplement: Supplementary file 1 — Supplementary Information [file 41467_2018_6237_MOESM1_ESM.pdf]

Paf1 and Ctr9 subcomplex formation is essential for Paf1 complex assembly and functional regulation

Xie et al.

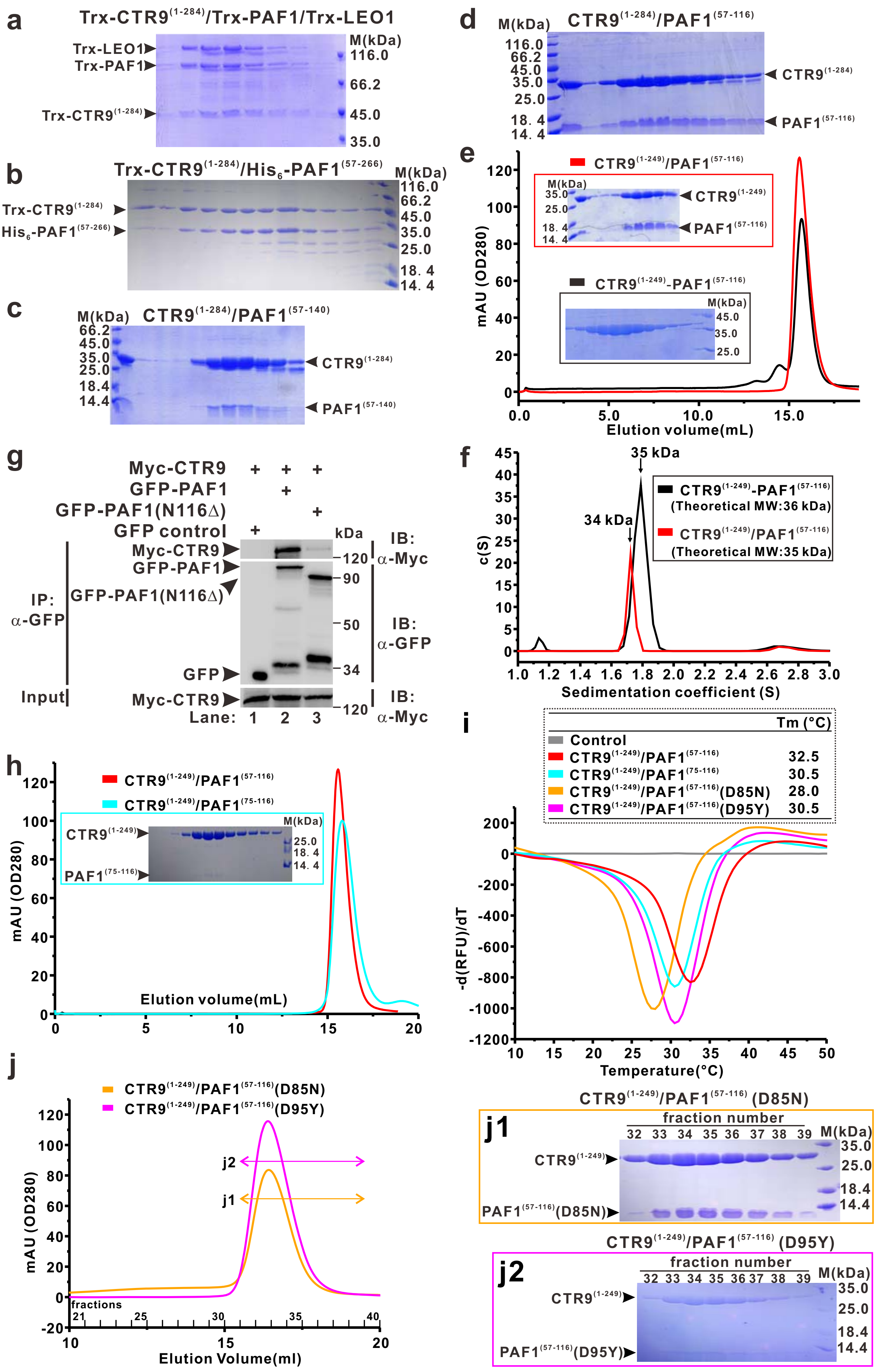

a
Trx-CTR9(1-284)/Trx-PAF1/Trx-LEO1
Trx-LEO1
Trx-PAF1
Trx-CTR9(1-284)
M(kDa)
116.0
66.2
45.0
35.0
b
Trx-CTR9(1-284)/His6-PAF1(57-266)
Trx-CTR9(1-284)
His6-PAF1(57-266)
c
CTR9(1-284)/PAF1(57-140)
CTR9(1-284)
PAF1(57-140)
d
CTR9(1-284)/PAF1(57-116)
CTR9(1-284)
PAF1(57-116)
e
CTR9(1-249)/PAF1(57-116)
CTR9(1-249)-PAF1(57-116)
mAU (OD280)
Elution volume(mL)
f
35 kDa
34 kDa
CTR9(1-249)-PAF1(57-116) (Theoretical MW:36 kDa)
CTR9(1-249)/PAF1(57-116) (Theoretical MW:35 kDa)
c(S)
Sedimentation coefficient (S)
g
Myc-CTR9
GFP-PAF1
GFP-PAF1(N116Δ)
GFP control
IP: α-GFP
Input
GFP
IB: α-Myc
IB: α-GFP
Lane: 1 2 3
h
CTR9(1-249)/PAF1(57-116)
CTR9(1-249)/PAF1(75-116)
PAF1(75-116)
Elution volume(mL)
i
Tm (°C)
Control
CTR9(1-249)/PAF1(57-116) 32.5
CTR9(1-249)/PAF1(75-116) 30.5
CTR9(1-249)/PAF1(57-116)(D85N) 28.0
CTR9(1-249)/PAF1(57-116)(D95Y) 30.5
-d(RFU)/dT
Temperature(°C)
j
CTR9(1-249)/PAF1(57-116)(D85N)
CTR9(1-249)/PAF1(57-116)(D95Y)
fractions
Elution Volume(ml)
j1
CTR9(1-249)/PAF1(57-116) (D85N)
fraction number
PAF1(57-116)(D85N)
j2
CTR9(1-249)/PAF1(57-116) (D95Y)
PAF1(57-116)(D95Y)

Supplementary Figure 1. Human CTR9/PAF1 subcomplex adopt heterodimer with a 1:1 stoichiometry. (**a**-**e**) SDS-PAGE of various human CTR9/PAF1 recombinant proteins by co-expression after gel filtration. For clarity, analytical gel filtration profiles are not shown, except two separate chains of CTR9$^{(1-249)}$/PAF1$^{(57-116)}$ complex protein and the single chain fusion of CTR9$^{(1-249)}$-PAF1$^{(57-116)}$ protein. "/" denotes protein complex with separate chains, while "-" denotes protein in a single-chain fusion (**e**). (**f**) The molecular weights of the purified proteins were measured by analytical ultracentrifugation sedimentation velocity (SV). c(s) distributions from the SV runs for the CTR9$^{(1-249)}$-PAF1$^{(57-116)}$ (1.4 mg/ml, black line) and CTR9$^{(1-249)}$/PAF1$^{(57-116)}$ (0.5 mg/ml, red line). (**g**) The N-terminal fragment amino acids 1-116 of PAF1 is essential for the interaction between full-length CTR9 and PAF1. Extracts were prepared from HEK293T cells transfected with various combinations of plasmids, as indicated. The bottom panel shows 3% of the Myc fusion proteins for each IP. (**h** and **j**) Analytical gel filtration profiles and SDS-PAGEs of CTR9$^{(1-249)}$/PAF1$^{(57-116)}$ (red line in **h**), CTR9$^{(1-249)}$/PAF1$^{(75-116)}$ (cyan line and insert in **h**), CTR9$^{(1-249)}$/PAF1$^{(57-116)}$(D85N) (**j** and **j1**) and CTR9$^{(1-249)}$/PAF1$^{(57-116)}$(D95Y) (**j** and **j2**). Those fractions shown in correspondent gels are indicated by a two-way arrow. (**i**) Differential scanning fluorimetry-based thermal denaturation assay showing the temperature-dependent denaturation profiles of CTR9$^{(1-249)}$/PAF1$^{(75-116)}$ and CTR9$^{(1-249)}$/PAF1$^{(57-116)}$ WT or mutants. Uncropped gels are shown in Supplementary Fig. 8.

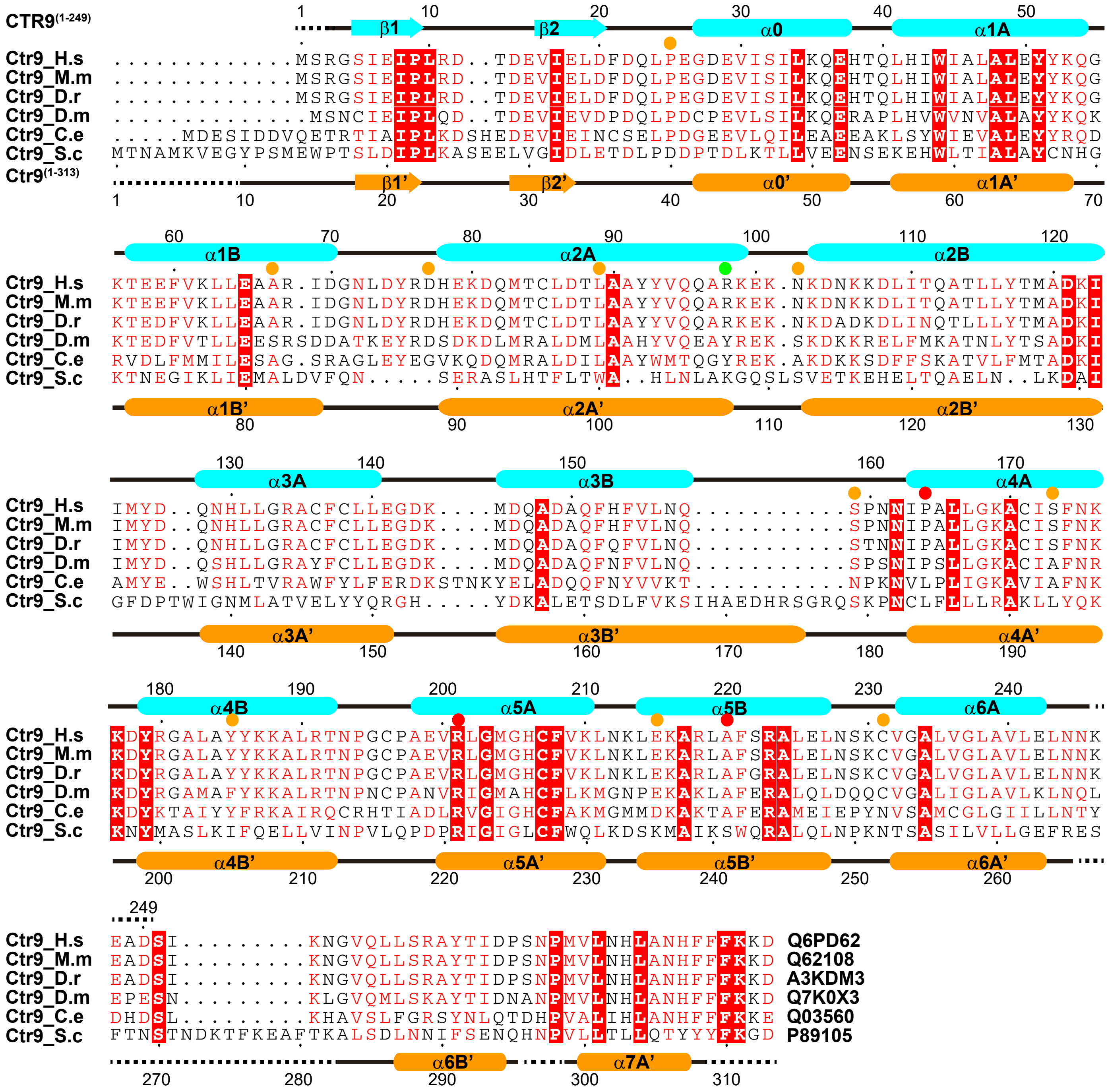

CTR9(1-249)
β1 β2 α0 α1A
Ctr9_H.s .............MSRGSIEIPLRD..TDEVIELDFDQLPEGDEVISILKQEHTQLHIWIALALEYYKQG
Ctr9_M.m .............MSRGSIEIPLRD..TDEVIELDFDQLPEGDEVISILKQEHTQLHIWIALALEYYKQG
Ctr9_D.r .............MSRGSIEIPLRD..TDEVIELDFDQLPEGDEVISILKQEHTQLHIWIALALEYYKQG
Ctr9_D.m ....MSNCIEIPLQD..TDEVIEVDPDQLPDCPEVLSILKQERAPLHVWVNVALAYYKQK
Ctr9_C.e .....MDESIDDVQETRTIAIPLKDSHEDEVIEINCSELPDGEEVLQILEAEEAKLSYWIEVALEYYRQD
Ctr9_S.c MTNAMKVEGYPSMEWPTSLDIPLKASEELVGIDLETDLPDDPTDLKTLLVEENSEKEHWLTIALAYCNHG
Ctr9(1-313)
β1' β2' α0' α1A'
α1B α2A α2B
Ctr9_H.s KTEEFVKLLEAAR.IDGNLDYRDHEKDQMTCLDTLAAYYVQQARKEK.NKDNKKDLITQATLLYTMADKI
Ctr9_M.m KTEEFVKLLEAAR.IDGNLDYRDHEKDQMTCLDTLAAYYVQQARKEK.NKDNKKDLITQATLLYTMADKI
Ctr9_D.r KTEDFVKLLEAAR.IDGNLDYRDHEKDQMTCLDTLAAYYVQQARKEK.NKDADKDLINQTLLLYTMADKI
Ctr9_D.m KTEDFVTLLEESRSDDATKEYRDSDKDLMRALDMLAAHYVQEAYREK.SKDKKRELFMKATNLYTSADKI
Ctr9_C.e RVDLFMMILESAG.SRAGLEYEGVKQDQMRALDILAAYWMTQGYREK.AKDKKSDFFSKATVLFMTADKI
Ctr9_S.c KTNEGIKLIEMALDVFQN.....SERASLHTFLTWA..HLNLAKGQSLSVETKEHELTQAELN..LKDAI
α1B' α2A' α2B'
α3A α3B α4A
Ctr9_H.s IMYD..QNHLLGRACFCLLEGDK....MDQADAQFHFVLNQ...........SPNNIPALLGKACISFNK
Ctr9_M.m IMYD..QNHLLGRACFCLLEGDK....MDQADAQFHFVLNQ...........SPNNIPALLGKACISFNK
Ctr9_D.r IMYD..QNHLLGRACFCLLEGDK....MDQADAQFQFVLNQ...........STNNIPALLGKACISFNK
Ctr9_D.m IMYD..QSHLLGRAYFCLLEGDK....MDQADAQFNFVLNQ...........SPSNIPSLLGKACIAFNR
Ctr9_C.e AMYE..WSHLTVRAWFYLFERDKSTNKYELADQQFNYVVKT...........NPKNVLPLIGKAVIAFNK
Ctr9_S.c GFDPTWIGNMLATVELYYQRGH.....YDKALETSDLFVKSIHAEDHRSGRQSKPNCLFLLLRAKLLYQK
α3A' α3B' α4A'
α4B α5A α5B α6A
Ctr9_H.s KDYRGALAYYKKALRTNPGCPAEVRLGMGHCFVKLNKLEKARLAFSRALELNSKCVGALVGLAVLELNNK
Ctr9_M.m KDYRGALAYYKKALRTNPGCPAEVRLGMGHCFVKLNKLEKARLAFSRALELNSKCVGALVGLAVLELNNK
Ctr9_D.r KDYRGALAYYKKALRTNPGCPAEVRLGMGHCFVKLNKLEKARLAFGRALELNSKCVGALVGLAVLELNNK
Ctr9_D.m KDYRGAMAFYKKALRTNPNCPANVRIGMAHCFLKMGNPEKAKLAFERALQLDQQCVGALIGLAVLKLNQL
Ctr9_C.e KDYKTAIYYFRKAIRQCRHTIADLRVGIGHCFAKMGMMDKAKTAFERAMEIEPYNVSAMCGLGIILLNTY
Ctr9_S.c KNYMASLKIFQELLVINPVLQPDPRIGIGLCFWQLKDSKMAIKSWQRALQLNPKNTSASILVLLGEFRES
α4B' α5A' α5B' α6A'
249
Ctr9_H.s EADSI.........KNGVQLLSRAYTIDPSNPMVLNHLANHFFFKKD Q6PD62
Ctr9_M.m EADSI.........KNGVQLLSRAYTIDPSNPMVLNHLANHFFFKKD Q62108
Ctr9_D.r EADSI.........KNGVQLLSRAYTIDPSNPMVLNHLANHFFFKKD A3KDM3
Ctr9_D.m EPESN.........KLGVQMLSKAYTIDNANPMVLNHLANHFFFKKD Q7K0X3
Ctr9_C.e DHDSL.........KHAVSLFGRSYNLQTDHPVALIHLANHFFFKKE Q03560
Ctr9_S.c FTNSTNDKTFKEAFTKALSDLNNIFSENQHNPVLLTLLQTYYYFKGD P89105
α6B' α7A'

Supplementary Figure 2. Structure-based sequence alignment of CTR9 from different species. In this alignment, the secondary structures of human CTR9 and yeast Ctr9 are shown at the top and bottom, respectively, according to the crystal structures of CTR9$^{(1-249)}$-PAF1$^{(57-116)}$ and Ctr9$^{(1-313)}$-Paf1$^{(34-103)}$, and conserved residues are shaded in red. The disease-associated amino acids substitutions P164S, R201C, or A220T (Category 1 shown in Fig. **3a**), P25L, A67T D77V, L89F, N102K, S159F, S173F, Y185C, E215K, or C231F (Category 2 shown in Supplementary Table 2), and R98W (Category 3 shown in Fig. **3a**) are indicated with red, orange , and green spheres, respectively. The GenBank numbers are shown at the end of each alignment.

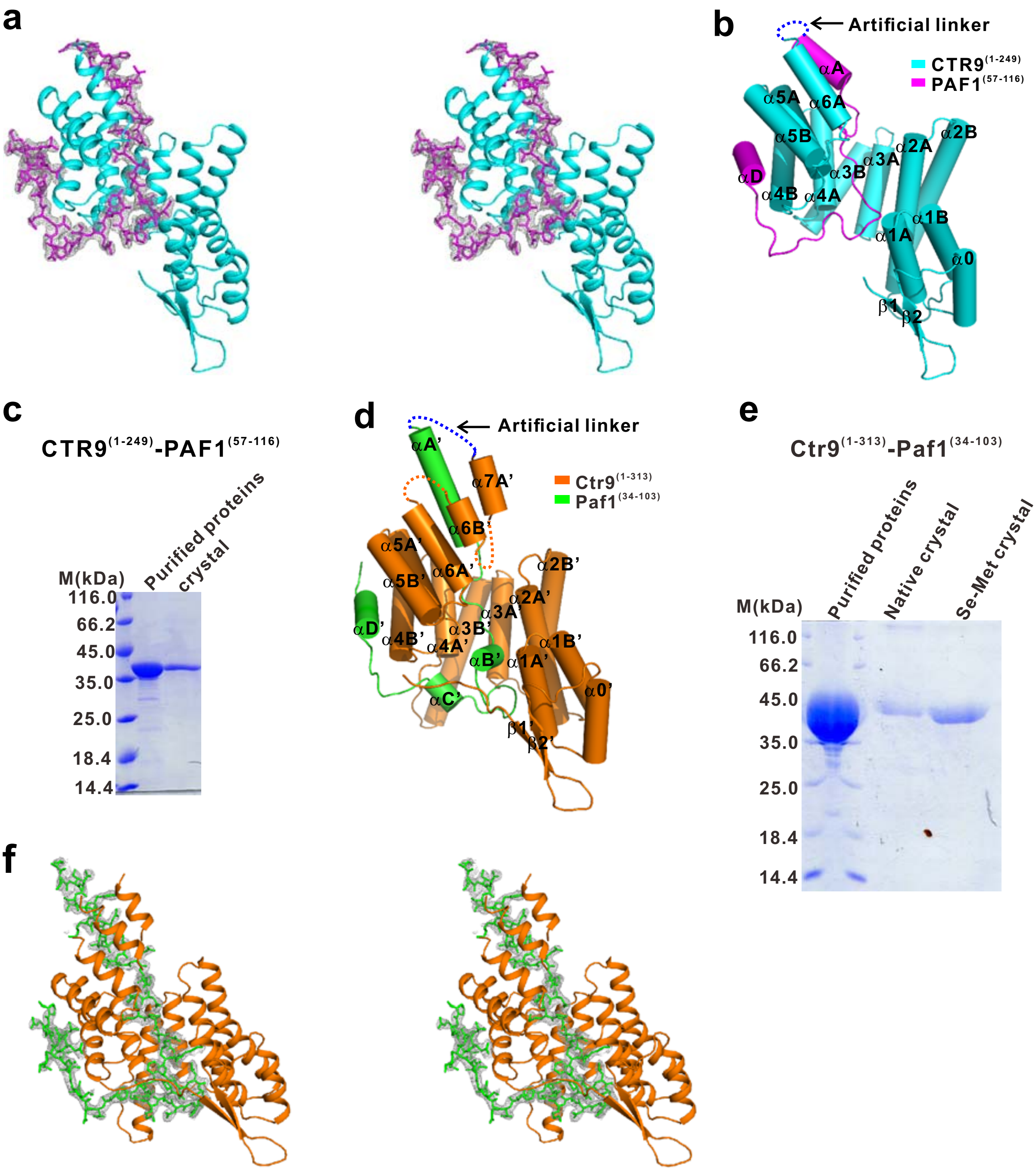
a
b
Artificial linker
CTR9(1-249)
PAF1(57-116)
αA
α5A
α6A
α5B
α2B
α2A
α3A
α3B
αD
α4B
α4A
α1B
α1A
α0
β1
β2
c
CTR9(1-249)-PAF1(57-116)
Purified proteins
crystal
M(kDa)
116.0
66.2
45.0
35.0
25.0
18.4
14.4
d
Artificial linker
αA'
α7A'
Ctr9(1-313)
Paf1(34-103)
α6B'
α5A'
α6A'
α2B'
α5B'
α2A'
α3A'
αD'
α3B'
α4B'
α4A'
α1B'
αB'
α1A'
α0'
αC'
β1'
β2'
e
Ctr9(1-313)-Paf1(34-103)
Purified proteins
Native crystal
Se-Met crystal
M(kDa)
116.0
66.2
45.0
35.0
25.0
18.4
14.4
f

Supplementary Figure 3. Comparison of purified and crystallized proteins. (**a** and **f**) Stereo images of the omit maps for the CTR9/PAF1 heteodimer (**a**) and the Ctr9/Paf1 heterodimer (**f**) contoured at 1.0 σ. (**b** and **d**) Cartoon representations of the overall structure of the single chain fusion of the CTR9 (cyan)/PAF1 (magenta) heterodimer (**b**) and the Ctr9 (orange)/Paf1 (green) heterodimer (**d**). (**c** and **e**) SDS-PAGEs of the Purified $CTR9^{(1-249)}$-$PAF1^{(57-116)}$ and the $CTR9^{(1-249)}$-$PAF1^{(57-116)}$ proteins dissolved from crystals are shown (**c**), and the Purified $Ctr9^{(1-313)}$-$Paf1^{(34-103)}$ and the $Ctr9^{(1-313)}$-$Paf1^{(34-103)}$ proteins dissolved from native or Se-Met crystals are shown (**e**). The crystals were transferred into mother liquor, washed three times and finally dissolved into 10 μl buffer (20 mM HEPES, pH 7.5, 150 mM NaCl, 1 mM EDTA, and 1 mM DTT). All solutions were loaded onto a 15% SDS-PAGE gel.

a

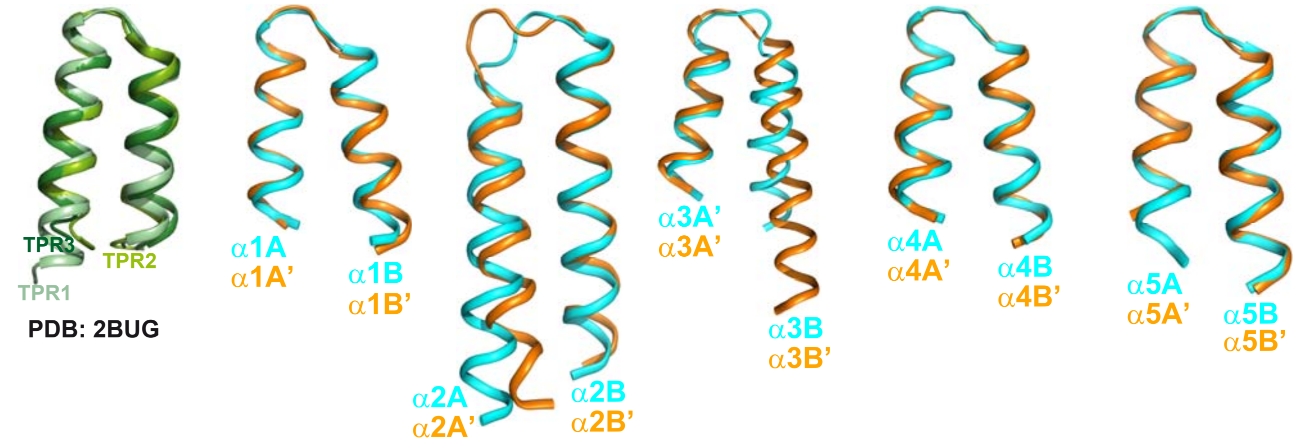

b

```
                          A helix                                 B helix
Canonical TPR         W4 L7G8 Y11                        A20  Y24 A27  P32
Human  TPR1  41  .....LHIWIALALEYYKQGK...........TEEFVKLLEAARIDGNLD........  74
       TPR2  78  HEKDQMTCLDTLAAYYVQQARKEKNKDNKKDLITQATLLYTMADKIIMYD........ 127
       TPR3 129  .....NHLLGRACFCLLEGDK...........MDQADAQFHFVLNQSPNN........ 162
       TPR4 163  .....IPALLGKACISFNKKD...........YRGALAYYKKALRTNPGC........ 196
       TPR5 198  .....AEVRLGMGHCFVKLNK...........LEKARLAFSRALELNSKC........ 231
Yeast  TPR1  56  .....KEHWLTIALAYCNHGK...........TNEGIKLIEMALDVFQNS........  89
       TPR2  90  ERASLHTFLTWAHLNLAKGQS..LSVETKEHELTQAELNLKDAIGFDPTW........ 137
       TPR3 138  ....IGNMLATVELYYQRGHY.........DKALETSDLFVKSIHAEDHRSGRQSKPN 182
       TPR4 183  .....CLFLLLRAKLLYQKKN...........YMASLKIFQELLVINPVL........ 216
       TPR5 220  .....PDPRIGIGLCFWQLKD...........SKMAIKSWQRALQLNPKN........ 252
```

c

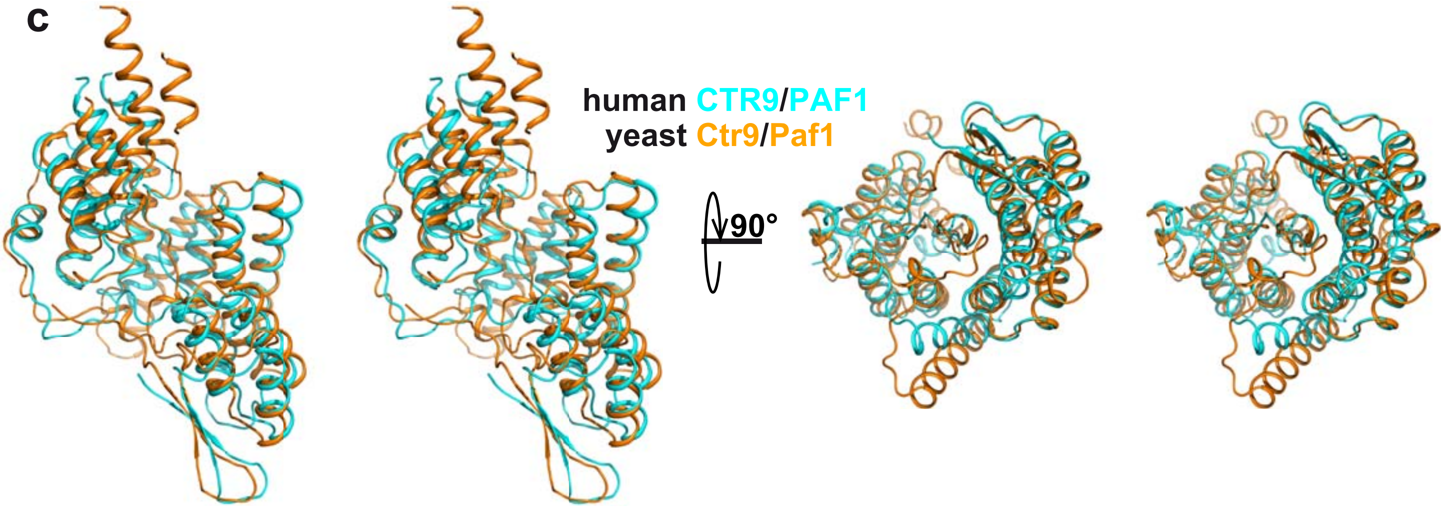

Supplementary Figure 4. Human CTR9/PAF1 and yeast Ctr9/Paf1 subcomplexes contain either canonical or atypical TPR motifs and have a similar heterodimeric structure. (**a**) Five TPR motifs of Ctr9 are compared with the canonical TPR fold of the Ppp5 protein. The motifs from human CTR9 and yeast Ctr9 are colored cyan and orange, respectively. (**b**) Structure-based sequence alignment of all 10 TPRs in human CTR9 and yeast Ctr9. The canonical TPR sequence is shown at the top cylinder (Lotus purple) and the conserved residues of Ctr9 are shaded in red. (**c**) Superimposition of the structures of the human CTR9/PAF1 (cyan) heterodimer and the yeast Ctr9/Paf1 (orange) heterodimer.

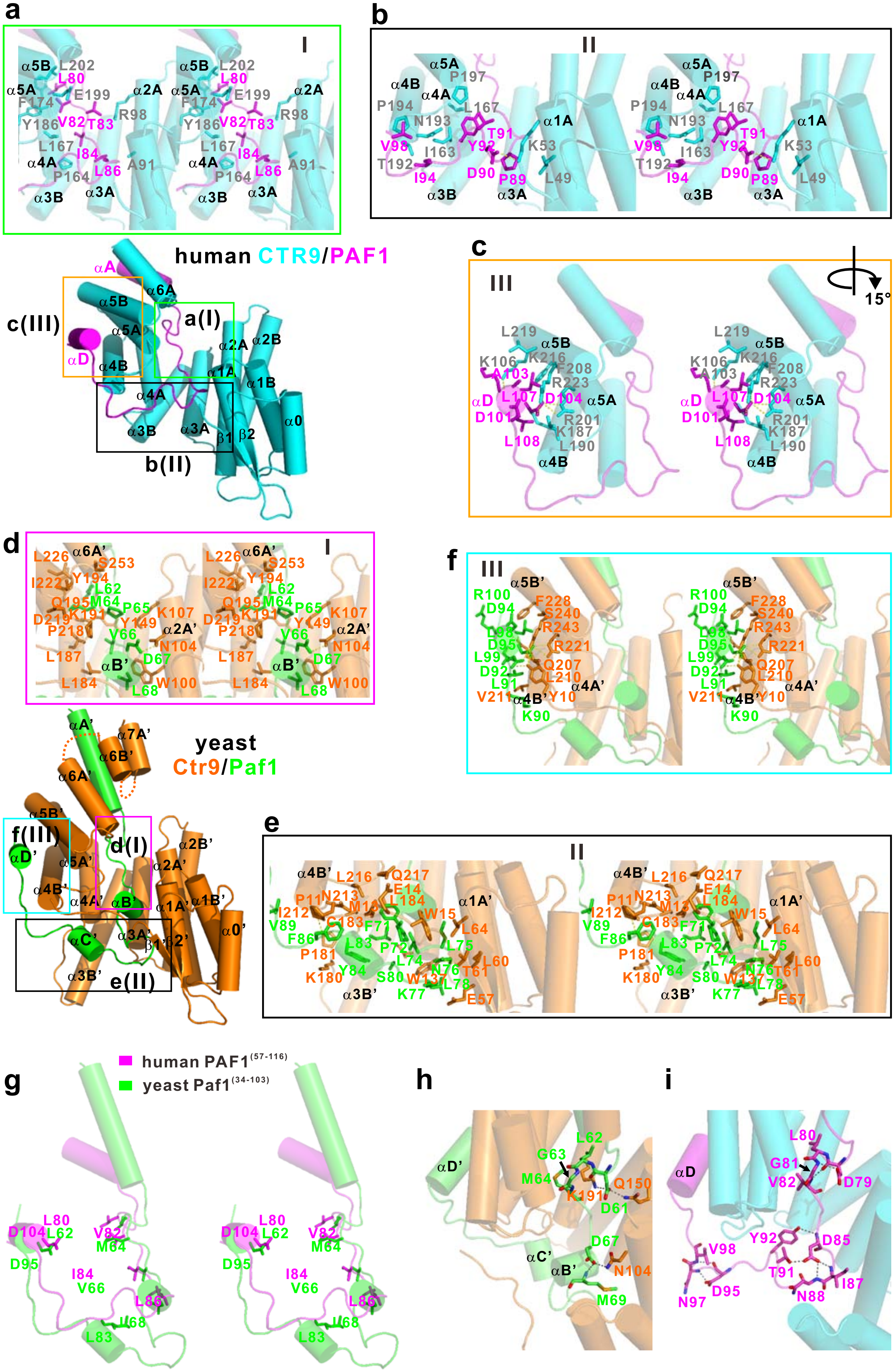

a
I
α5B L202
L80
α5A E199 α2A
F174
Y186 V82 T83 R98
L167
I84
α4A L86 A91
P164
α3B α3A
b
II
α5A
α4B P197
α4A
P194 L167
N193 α1A
T91
V98 I163 Y92 K53
T192
I94 D90 P89 L49
α3B α3A
human CTR9/PAF1
αA
α6A
α5B
c(III) a(I)
α5A
α2A α2B
αD
α4B α1A
α1B
α4A
α0
α3B α3A
β1 β2
b(II)
c
III
15°
L219
α5B
K106 K216
A103 F208
R223
αD L107 D104 α5A
D101 R201
K187
L108 L190
α4B
d
I
L226 α6A'
S253
I222 Y194
L62
Q195 M64 P65
K107
D219 K191
Y149
P218 α2A'
V66
N104
L187
αB' D67
L184 W100
L68
f
III
α5B'
R100 F228
D94 S240
R243
D98
D95 R221
L99
Q207
D92 L210
L91 α4A'
V211 α4B' Y10
K90
yeast Ctr9/Paf1
αA'
α7A'
α6B'
α6A'
α5B'
f(III) d(I)
αD'
α2B'
α5A'
α2A'
α4B'
α4A' αB' α1A' α1B'
αC' α3A'
α0'
β1' β2'
α3B' e(II)
e
II
α4B' L216 Q217
E14
P11 N213
M13 L184 α1A'
I212
W15
V89 C183 F71
L64
F86
L83 P72 L75
P181
L74 L60
Y84 N76 T61
K180 S80 W137
L78
α3B' K77 E57
g
human PAF1(57-116)
yeast Paf1(34-103)
L80
D104 L62 V82
M64
D95
I84
V66 L86
L68
L83
h
L62
αD'
G63
M64 Q150
K191
D61
D67
αC'
αB' N104
M69
i
L80
αD
G81
V82 D79
Y92 D85
V98
T91
I87
N97 D95 N88

Supplementary Figure 5. The interaction interface of the human CTR9/PAF1 and yeast Ctr9/Paf1 heterodimers. (**a-b**) The CTR9$^{(1-249)}$/PAF1$^{(57-116)}$ interface is divided into three regions corresponding to the CTR9 TPR1-5 concave channel/N-terminal loop of PAF1 (**a**), the side of the TPR1-5/C-terminal loop of PAF1 (**b**), and the convex surface of the TPR4-5/αD helix of PAF1 (**c**). (**d-f**) The Ctr9$^{(1-313)}$/Paf1$^{(34-103)}$ interface is divided into three regions corresponding to the Ctr9 TPR1-5 concave channel/N-terminal loop of Paf1 (**d**), the side of the TPR1-5/C-terminal loop of Paf1 (**e**), and the convex surface of the TPR4-5/αD' helix of Paf1 (**f**). The interaction details between CTR9 and PAF1 (**a-c**) or between Ctr9 and Paf1 (**d-f**) in the three regions are shown in stereo view. The side chain of the residues involved in the interactions are drown in the stick model. Charge-charge and hydrogen bonding interactions are highlighted by dashed yellow lines. (**g**) A stereo view of the superimposition of the hook-fold structures of PAF1$^{(57-116)}$ and Paf1$^{(34-103)}$. PAF1 and Paf1 are colored magenta and green, respectively. (**h** and **i**) The highly conserved residues are important for yeast Ctr9/Paf1 (**h**) and human CTR9/PAF1 (**i**) complexes formation. For clarify, only residues involved in hydrogen bonding are shown in (**h**) and (**i**), and the side chain are drawn in the stick model. Hydrogen bonding interactions are highlighted by dashed grey lines.

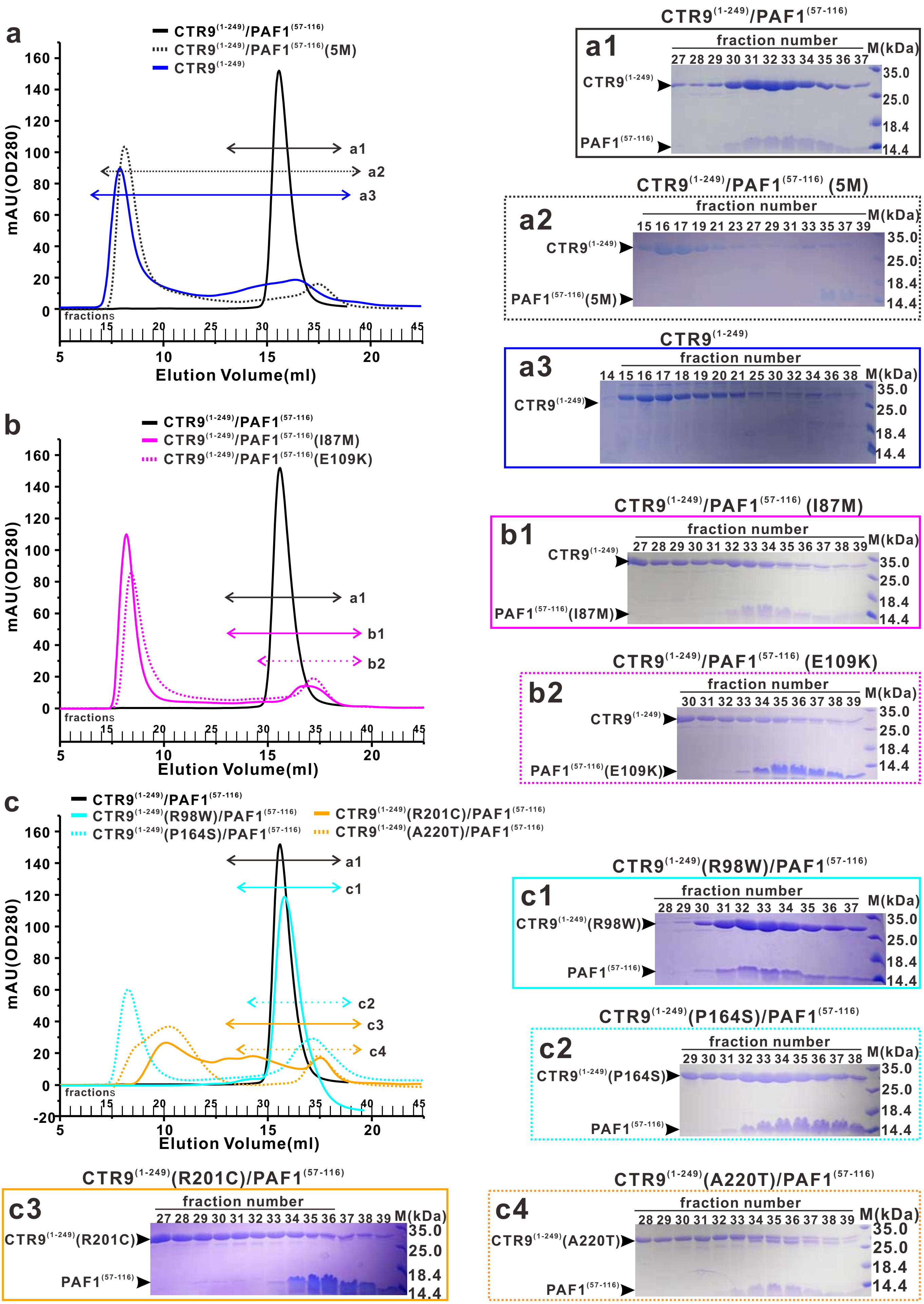
a
CTR9(1-249)/PAF1(57-116)
CTR9(1-249)/PAF1(57-116)(5M)
CTR9(1-249)
mAU(OD280)
Elution Volume(ml)
fractions
a1
a2
a3
CTR9(1-249)/PAF1(57-116)
fraction number
M(kDa)
27 28 29 30 31 32 33 34 35 36 37
CTR9(1-249)
PAF1(57-116)
35.0
25.0
18.4
14.4
CTR9(1-249)/PAF1(57-116) (5M)
15 16 17 19 21 23 27 29 31 33 35 37 39
PAF1(57-116)(5M)
CTR9(1-249)
14 15 16 17 18 19 20 21 25 30 32 34 36 38
b
CTR9(1-249)/PAF1(57-116)(I87M)
CTR9(1-249)/PAF1(57-116)(E109K)
b1
b2
27 28 29 30 31 32 33 34 35 36 37 38 39
PAF1(57-116)(I87M)
30 31 32 33 34 35 36 37 38 39
PAF1(57-116)(E109K)
c
CTR9(1-249)(R98W)/PAF1(57-116)
CTR9(1-249)(P164S)/PAF1(57-116)
CTR9(1-249)(R201C)/PAF1(57-116)
CTR9(1-249)(A220T)/PAF1(57-116)
c1
c2
c3
c4
28 29 30 31 32 33 34 35 36 37
CTR9(1-249)(R98W)
29 30 31 32 33 34 35 36 37 38
CTR9(1-249)(P164S)
27 28 29 30 31 32 33 34 35 36 37 38 39
CTR9(1-249)(R201C)
28 29 30 31 32 33 34 35 36 37 38 39
CTR9(1-249)(A220T)

Supplementary Figure 6. Disease-associated amino acid substitutions abolish the interactions between CTR9$^{(1-249)}$ and PAF1$^{(57-116)}$. (**a**-**c**) Analytical gel filtration profiles and SDS-PAGEs of CTR9$^{(1-249)}$/PAF1$^{(57-116)}$ (**a** and **a1**), CTR9$^{(1-249)}$/PAF1$^{(57-116)}$(5M) (**a** and **a2**), CTR9$^{(1-249)}$ (**a** and **a3**), CTR9$^{(1-249)}$/PAF1$^{(57-116)}$(I87M) (**b** and **b1**), CTR9$^{(1-249)}$/PAF1$^{(57-116)}$(E109K) (**b** and **b2**), CTR9$^{(1-249)}$(R98W)/PAF1$^{(57-116)}$ (**c** and **c1**), CTR9$^{(1-249)}$(P164S)/PAF1$^{(57-116)}$ (**c** and **c2**), CTR9$^{(1-249)}$(R201C)/PAF1$^{(57-116)}$ (**c** and **c3**), and CTR9$^{(1-249)}$(A220T)/PAF1$^{(57-116)}$ (**c** and **c4**). Those fractions shown in correspondent gels are indicated by a two-way arrow. Uncropped gels are shown in Supplementary Fig. 8.

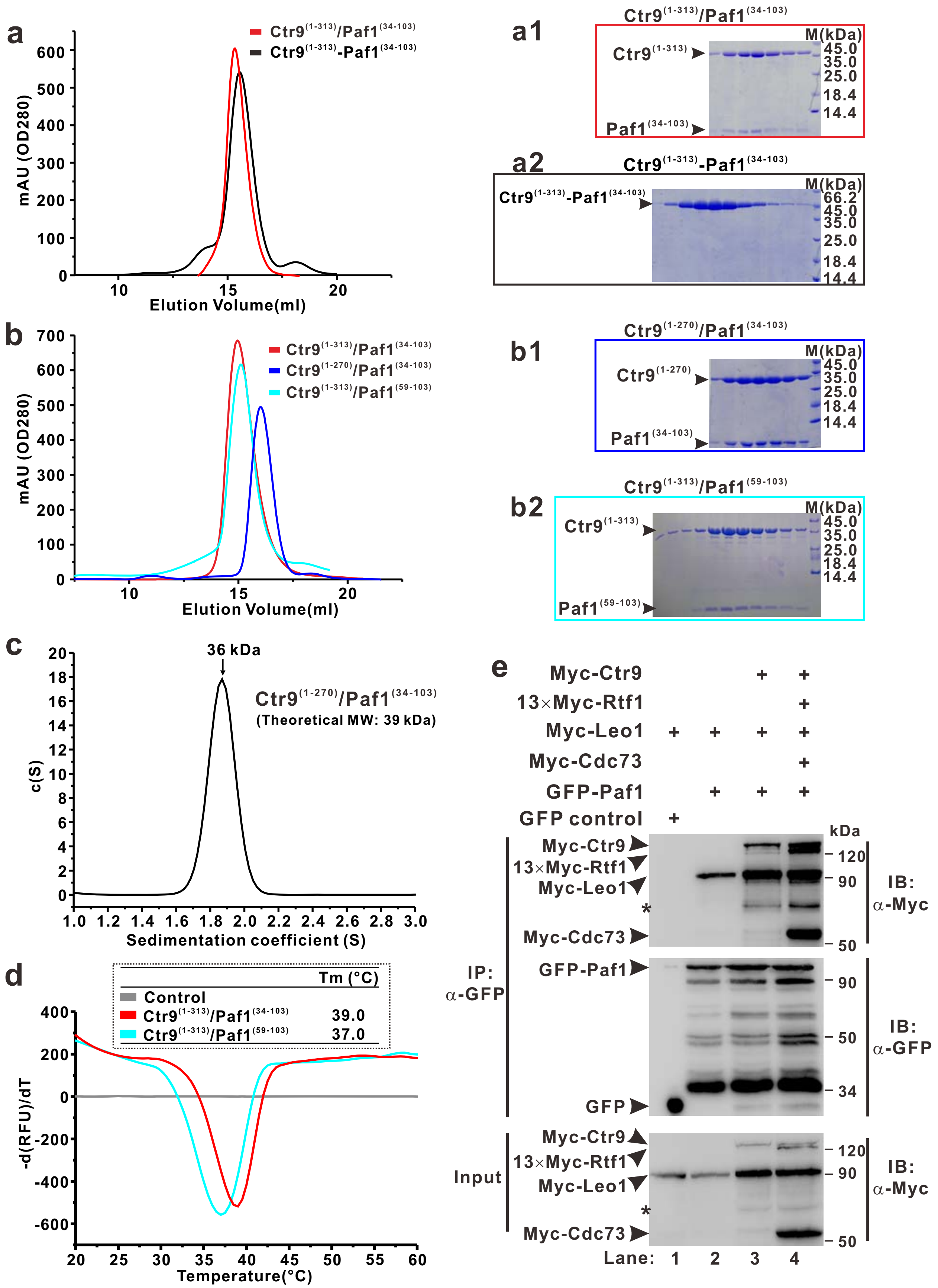
a
Ctr9(1-313)/Paf1(34-103)
Ctr9(1-313)-Paf1(34-103)
mAU (OD280)
Elution Volume(ml)
a1
Ctr9(1-313)/Paf1(34-103)
M(kDa)
Ctr9(1-313)
Paf1(34-103)
45.0
35.0
25.0
18.4
14.4
a2
Ctr9(1-313)-Paf1(34-103)
M(kDa)
66.2
45.0
35.0
25.0
18.4
14.4
b
Ctr9(1-313)/Paf1(34-103)
Ctr9(1-270)/Paf1(34-103)
Ctr9(1-313)/Paf1(59-103)
mAU (OD280)
Elution Volume(ml)
b1
Ctr9(1-270)/Paf1(34-103)
M(kDa)
Ctr9(1-270)
Paf1(34-103)
b2
Ctr9(1-313)/Paf1(59-103)
M(kDa)
Ctr9(1-313)
Paf1(59-103)
c
36 kDa
Ctr9(1-270)/Paf1(34-103)
(Theoretical MW: 39 kDa)
c(S)
Sedimentation coefficient (S)
d
Tm (°C)
Control
Ctr9(1-313)/Paf1(34-103) 39.0
Ctr9(1-313)/Paf1(59-103) 37.0
-d(RFU)/dT
Temperature(°C)
e
Myc-Ctr9
13×Myc-Rtf1
Myc-Leo1
Myc-Cdc73
GFP-Paf1
GFP control
kDa
IP: α-GFP
IB: α-Myc
IB: α-GFP
GFP
Input
Lane: 1 2 3 4

Supplementary Figure 7. Yeast Ctr9/Paf1 subcomplexes adopt heterodimers with a 1:1 stoichiometry. (**a** and **b**) Analytical gel filtration profiles and SDS-PAGE of two separate chains of Ctr9$^{(1-313)}$/Paf1$^{(34-103)}$ complex protein (**a** and **a1**) and the single chain fusion of Ctr9$^{(1-313)}$-Paf1$^{(34-103)}$ protein (**a** and **a2**). "/" denotes protein complex with separate chains, while "-" denotes protein in a single-chain fusion. Analytical gel filtration profiles and SDS-PAGEs of Ctr9$^{(1-270)}$/Paf1$^{(34-103)}$ (**b** and **b1**) and Ctr9$^{(1-270)}$/Paf1$^{(59-103)}$ (**b** and **b2**). (**c**) c(s) distributions from the SV run for the Ctr9$^{(1-270)}$/Paf1$^{(34-103)}$ (1 mg/ml). (**d**) Differential scanning fluorimetry-based thermal denaturation assay showing the temperature-dependent denaturation profiles of Ctr9$^{(1-313)}$/Paf1$^{(34-103)}$ (red line) and Ctr9$^{(1-313)}$/Paf1$^{(59-103)}$ (cyan line). (**e**) Co-IP experiments by GFP-tagged yeast Paf1 (GFP-Paf1). Extracts were prepared from HEK293T cells transfected with various combinations of plasmids, as indicated, immunoprecipitated with agarose-conjugated anti-GFP and subsequently immunoblotted with anti-Myc or anti-GFP, as indicated. The top panel shows the IP results. The middle panel represents the IP of GFP and GFP-Paf1. The bottom panel shows 3% input of the Myc fusion proteins used for each IP. The asterisk indicate the degradation of Myc-Ctr9. Uncropped gels and blots are shown in Supplementary Fig. 8.

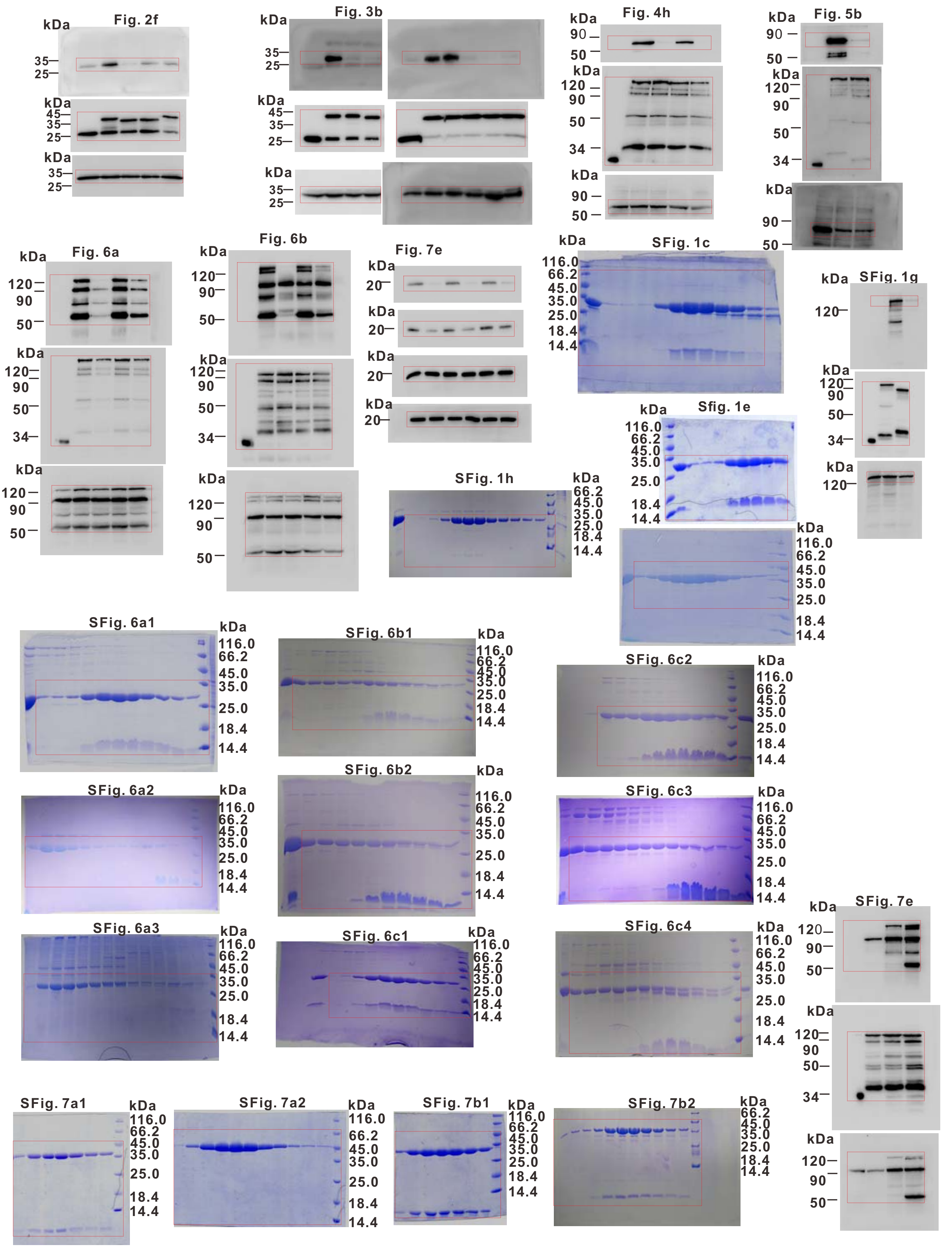

Supplementary Figure 8. Full uncropped figures of western blots and SDS-PAGE gels. Cropped regions are indicated with rectangles as appropriate.

Supplementary Table 1. Data collection and refinement statistics

	Ctr9-Paf1 Se-Met	CTR9-PAF1 Se-Met	CTR9-PAF1 Native
**Data collection**			
Space group	***P***3221	***P***3112	***P***3112
Cell dimensions			
a, *b*, *c* (Å)	95.95, 95.95, 115.50	82.55, 82.55, 145.54	82.42, 82.42, 145.66
α, β, γ (°)	90, 90, 120	90, 90, 120	90, 90, 120
Resolution (Å)	50.00-2.53 (2.62-2.53) [a]	50.00-2.83 (2.88-2.83) [a]	50.00-2.53 (2.57-2.53) [a]
R_{merge} (%)	4.7 (85.8)	7.3 (67.0)	6.7 (63.2)
$I / \sigma I$	29.2 (2.0)	38.8 (4.0)	28.9 (4.0)
Completeness (%)	99.8 (99.9)	100.0 (99.6)	100.0 (100.0)
Redundancy	5.5 (5.5)	19.7(16.6)	9.9 (10.1)
**Refinement**			
Resolution (Å)	47.98-2.53		41.21-2.53
No. reflections	35,677		17,815
R_{work} / R_{free}	19.39/24.30		20.18/24.02
No. atoms			
Protein	2,739		2,276
Ligand/ion	3		0
Water	85		103
B-factors			
Protein	47.6		55.7
Ligand/ion	53.5		
Water	39.7		44.9
R.m.s. deviations			
Bond lengths (Å)	0.008		0.009
Bond angles (°)	0.984		1.078

[a] Values in parentheses are for highest-resolution shell.

Supplementary Table 2. Disease-associated mutations in human CTR9

Mutation type	Mutation (Amino acid)
Missence	L10F, E15G, L19I, P25L, Q36R, I43V, A46D, A48V, L63V, A67T, D74H, D77V, L89F, R98W, N102K, D108V, T118A, D121N, Y126C, N129I, H153Y, N157S, S159F, P164S, K169N, S173F, Y185C, K188N, R191H, A198T, R201C, K213T, E215K, A220T, A224S, E226K, C231F, V232M, S250F, S267N, L272W, A276E, V288F, H294Y, M304K, E320G, Y329C, F344C, E376K, G382S, A386T, G399C, H400Y, K402E, D426N, A437T, P450T, L454F, N456D, A459V, G468W, R480S, R480C, A494T, A494S, A494P, A494V, A509V, R525L, E526K, Y534C, R536H, E556A, W568L, G572D, G572V, N573H, W581C, P583H, S596F, G608S, Q613K, R620Q, K624N, R627H, R631H, R631C, K637R, R641K, K645M, N646K, L657V, H659P, R664C, R667C, T677S, A678T, D682V, A688V, A700T, Y704C, Y704D, H714D, V720I, Y722C, A747T, D750N, L764F, D771N, A782V, A789V, Y792N, L796F, V799A, V799L, G800E, D806H, S818F, L820V, R830Q, R830W, R832H, D835Y, E838Q, R839Q, R839W, E846Q, K849N, R853M, L857P, Q860H, R864H, K872N, L874P, R878Q, R878W, E883K, L889I, M890T, F891C, R903G, R910H, R910C, S911C, S911F, E916D, D924Y, D926G, E948D, R961I, G966R, K982Q, R984Q, R985C, P986T, K993R, R999C, P1002L, S1003L, K1007R, S1015L, D1018H, D1022N, L1026H, K1027R, S1059F, N1062S, G1067D, E1069K, G1071S, R1075K, R1099Q, R1080W, P1090S, S1097Y, E1105K, R1122H, S1143Y, N1157I, S1161L, S1165L, D1173N
Nonsense	G27*, Q391*, R394*, E515*, E526*, W553*, Q629*, G654*, G800*, R1077*
Frameshift deletion	T57fs*5, K107fs*96, L475fs*63, V673fs*13, R903fs*102, D923fs*1
Frameshift insertion	L24fs*6 , L368Ffs*6
In-frame deletion	R962delR

Various disease-associated mutations in CTR9 were listed, based on the data extracted from the COSMIC database (http://cancer.sanger.ac.uk/cosmic). A total of 38 missense mutations were located in 38 residues of CTR9$^{(1\text{-}249)}$. These 38 mutations were divided into three categories. The three categories were named as interface, folding, and others, and amino acid substitutions in each category were colored in red, orange, and green, respectively. Only mutations used in this study were shown in Fig. 3a.

Supplementary Table 3. Disease-associated mutations in human PAF1

Mutation type	Mutation (Amino acid)
Missence	R11W, R11L, S38I, D54N, H70Q, K71R, H72N, H72R, H72Q, D73H, D73V, L74I, L74R, T76S, D85N, L86F, I87M, D95N, D95Y, E105D, E109K, A125T, K133N, T134I, E149K, E167K, I176L, P201L, E203D, M250V, D251N, G254R, F261S, E266K, T267M, R271Q, R273Q, E277K, D285N, W297L, N301H, G306D, G318C, R331C, R335H, R336Q, S343L, A347T, R354W, M356T, E358K, K359T, R367W, R367Q, P376L, E412Q, R420W, R420Q, K431R, K431N, E438K, E441K, R445Q, E469K, D470N, R471S, Q475L, D481A, G489A, R492W, S495N, A519V, D522N, A526D, D527N, D527H, D531N
Nonsense	E25*, G28*, W130*, R132*, R198*, E469*, G472*
Frameshift deletion	T76fs*24, S174fs*48, K230fs*11, Q475fs*>57, G485fs*>47
Frameshift insertion	G401fs*>134, G408fs*>125
In-frame deletion	F313delF, E399delE, K406delK, A519_A526delAASDSS...

Various disease-associated mutations in PAF1 were listed, based on the data extracted from the COSMIC database (http://cancer.sanger.ac.uk/cosmic). A total of 17 missense mutations were located in 12 residues of PAF[57-116]. These 17 mutations were divided into three categories. The three categories were named as interface, folding, and others, and amino acid substitutions in each category were colored in red, orange, and green, respectively. Only mutations used in this study were shown in Fig. 3a.

Supplementary Table 4. Yeast strains used in this study.

Strain name	Genotype	Reference
SEY6210	*MATalpha his3D200 leu2–3,112 lys2–801 trp1-D901 ura3–52 suc2-D9 GAL+*	1
YXY01	SEY6210 *URA3 LEU2*	This study
YXY02	SEY6210 *paf1::URA3*	This study
YXY03	SEY6210 *paf1::URA3 pP1k-PAF1::LEU2*	This study
YXY04	SEY6210 *paf1::URA3 pP1k-PAF1(4S)::LEU2*	This study
YXY05	SEY6210 *paf1::URA3 pP1k-PAF1(L83S)::LEU2*	This study
YXY06	SEY6210 *paf1::URA3 pP1k-PAF1(D95K)::LEU2*	This study
P	promoter	

Supplementary Reference:

1. Robinson JS, Klionsky DJ, Banta LM, Emr SD. Protein sorting in Saccharomyces cerevisiae: isolation of mutants defective in the delivery and processing of multiple vacuolar hydrolases. *Mol. Cell. Biol.* **8**, 4936-4948 (1988).
